# Assessment of Post-Dengue Rheumatic Symptoms Using the WOMAC and DAS-28 Questionnaires in a Honduran Population after a Four-Month Follow-Up

**DOI:** 10.3390/tropicalmed7120394

**Published:** 2022-11-23

**Authors:** Lysien I. Zambrano, Itzel Carolina Fuentes-Barahona, Ricardo Portillo-Pineda, Melissa Aguilar-Ponce, José Carlos Murillo-Padilla, Marlen Suazo-Menocal, Cesar Antunez-Salgado, Edissa Medina-Bassilet, Fausto Muñoz-Lara, D. Katterine Bonilla-Aldana, Juan J. Montenegro-Idrogo, Alfonso J. Rodríguez-Morales

**Affiliations:** 1 Unit of Scientific Research, School of Medicine, Faculty of Medical Sciences, Universidad Nacional Autónoma de Honduras (UNAH), Tegucigalpa 11101, Honduras; 2 Medical Faculty of the National Autonomous University of Honduras, Tegucigalpa 11101, Honduras; 3 Department of Gynecology and Obstetrics, Hospital Escuela, Tegucigalpa 11101, Honduras; 4 Faculty of Medicine, Catholic University of Honduras, Tegucigalpa 11101, Honduras; 5 Department of Internal Medicine, Faculty of Medical Sciences, Universidad Nacional Autónoma de Honduras (UNAH), Tegucigalpa 11101, Honduras; 6 Department of Internal Medicine, Hospital Escuela, Tegucigalpa 11101, Honduras; 7 Unit of Research, Universidad Continental, Huancayo 12001, Peru; 8 Service of Infectious and Tropical Diseases, Hospital Nacional Dos de Mayo, Lima 150101, Peru; 9 Master in Clinical Epidemiology and Biostatistics, Universidad Científica del Sur, Lima 150142, Peru; 10 Grupo de Investigación Biomedicina, Faculty of Medicine, Fundación Universitaria Autónoma de las Americas, Pereira 660003, Risaralda, Colombia; 11 Gilbert and Rose-Marie Chagoury School of Medicine, Lebanese American University, Beirut 1401, Lebanon

**Keywords:** arthritis, arbovirus infections, dengue, joint, arthralgia, Honduras

## Abstract

Introduction: Alphaviruses may cause arthritis, but there is a lack of studies assessing it in flaviviruses such as dengue. Through the 28 Joint Disease Activity Score (DAS-28), incorporating swollen joint counts, and through the Arthritis Index from Western Ontario and McMaster Universities (WOMAC), we assessed pain, stiffness, and dimensions of arthritic function in post-DENV patients. Methods: Prospective study of a cohort of participants who were diagnosed with dengue in centres in Honduras from December 2019 to February 2020, with a follow-up period of 4 months to evaluate post-dengue rheumatological disease through the WOMAC and DAS-28 questionnaires. Results: After a four-month follow-up phase with 281 participants, the final cohort comprised 58.8% women and 41.20% men. After the follow-up, 63.02% persisted with the clinical findings. According to WOMAC, joint involvement was higher in women with (58.76%) (*p* < 0.0001) these symptoms or functional limitations when performing daily activities were limited to pain when walking (34.81% vs. 5.51%), climbing or descending stairs (36.46% vs. 8.66%), and at night at bedtime (28.73% vs. 7.08%). With the DAS-28, we found at least one alteration with inflammation or pain in 14.91% of the participants, primarily women (*p* < 0.01). Discussion: Joint involvement was high during the dengue epidemic in 2019. We observed a significant proportion of women with inflammation and joint pain, showing that dengue may lead to the development of chronic rheumatological findings, although lower than in CHIKV, still affecting everyday life and, consequently, their quality of life. Additional long-term evaluation studies after dengue are required.

## 1. Introduction

Dengue (DENV) continues to be the most important viral vector-borne disease with large morbidity and mortality in Latin America and South-East Asia. Dengue is an arthropod-borne viral disease caused by four dengue virus serotypes (DENV 1-4) transmitted by species of *Aedes* mosquitoes, particularly *A. aegypti, A. albopictus,* and *A. vittatus*. Dengue is endemic in more than 100 countries in Southeast Asia, the Western Pacific, Africa, the eastern Mediterranean regions, and the tropical Americas. Additionally, autochthonous occurrences in southern Europe are a consequence of climate change. Its incidence has multiplied in the last 50 years [1]. In 2017, there were 580,640 dengue cases (254,453 in Brazil, 89,893 in Mexico, and 76,093 in Peru), with 561,356 in 2018 (265,934 from Brazil, 78,621 from Mexico, and 44,825 from Colombia). According to WHO, in 2020, they reported 2,300,558 cases in the Americas region [1]. In 2022, up to November 7, 2022, 2,499,358 cases had been reported in the Americas, with an incidence of 252.00 cases per 100,000 pop., 3641 cases of severe dengue (0.1%), and 1135 deaths for a case fatality rate of 0.045% (www.paho.org (accessed on 8 November 2022)).

In the case of Honduras, a Central American country with environmental and social conditions, is prone to vector-borne diseases, including those caused by arboviruses, such as DENV [2,3]. Factors such as temperature, vector bionomics (survival, density, frequency, and feeding behaviour), extrinsic incubation period, and vector competence can affect DENV transmission [4].

Multiple arboviruses, primarily alphaviruses, are related to chronic rheumatic manifestations, especially chikungunya [5,6]. Other alphaviruses, such as Mayaro and o’nyong-nyong viruses, may produce chronic rheumatic consequences [7,8]. The Mayaro virus is an arthritic alphavirus; the infection leads to a disease such as DENV; and there is the susceptibility of human chondrocytes, fibroblast-like synoviocytes, and osteoblasts, which are the main types of cells involved in osteoarthritis [9]. Viral load accumulates to the point that generalized clinical symptoms (fever, headache, and myalgia) develop, presumably secondary to an antiviral state of the host in which the expression of interferon is abundant [10]. Furthermore, arboviral infections are frequent causes of febrile illness [11]. In addition to alphaviruses, flaviviruses, including DENV, may also produce acute and non-acute rheumatic manifestations [12,13], although they have been poorly studied.

DENV virus infection can lead to asymptomatic or symptomatic infection. Approximately 20% of all patients are symptomatic, and individuals experience symptoms of the disease that cover a broad clinical spectrum from non-severe to severe clinical manifestations. The disease caused by DENV has an abrupt onset with three widely identifiable phases: febrile, critical, and recovery [14]. Typical symptoms include a fever that can exceed 39 °C and polyarthralgia. Symmetric bilateral arthralgia is found in most patients and is usually located in the peripheral joints, appearing shortly after the onset of fever (2 to 5 days) [15]. Arthritis is defined as pain with swelling of the joints, while arthralgia often refers to joint pain. However, the use of these terms is often not well defined.

Rheumatic diseases can contribute to a severe course of dengue, although their contribution has not been previously characterized [16]. Chikungunya is a disease, different from dengue, that would progress to a chronic phase, mainly producing chronic inflammatory rheumatism in about half of the patients; according to recent estimates and measurements in Latin America, co-infections between dengue and Chikungunya can occur and have already been reported [3]. A study in Tolima showed that approximately half of the patients persisted with post-CHIK arthralgia (arbovirus) after 24 weeks of follow-up [17]. In Chikungunya, possible causes of chronicity in arthralgia are viral persistence, genetic predisposition, induction of autoimmune diseases, tissue damage caused directly by the virus, and exacerbation of a pre-existing joint condition [18]. Chronic musculoskeletal symptoms, which are likely to be mediated by inflammation potentially resulting from viral persistence, may reappear or persist for more than three months after the acute phase of the disease [19].

The 28 Joint Disease Activity Score (DAS-28) is essential for clinical practice and research, incorporating swollen and sensitive joint counts [20]. The Arthritis Index from the Universities of Western Ontario and McMaster (WOMAC) is a self-administered health status measure that assesses pain, stiffness, and function dimensions. It produces three subscale scores (pain, stiffness, and physical function) and one total score (WOMAC index) that reflects disability in general [21].

The present study was carried out in two hospitals with Dengue units located in the northern part of Honduras, in the department of Atlántida (“Hospital General Atlántida”) and in Yoro (“Hospital Manuel de Jesus Subirana”), in conjunction with an intermediate patient care centre (“Centro Santiago Apostol Health Department”). We were wondering if the participants diagnosed with dengue during the 2019 epidemic presented joint involvement by completing the evaluation questionnaires for rheumatic diseases in the WOMAC and DAS-28.

## 2. Methods

The present research is a prospective study of a cohort of participants who were diagnosed with dengue in the emergency unit or dengue unit of the General Hospital Atlántida, the Santiago Apostol Comprehensive Health Center in Yoro, and the Manuel de Jesús Subirana Hospital in Yoro in the period from December 2019 to February 2020, which corresponds to Phase I, and from March to June 2020, which corresponds to Phase II.

### 2.1. Inclusion Criteria

**Figure 1:**
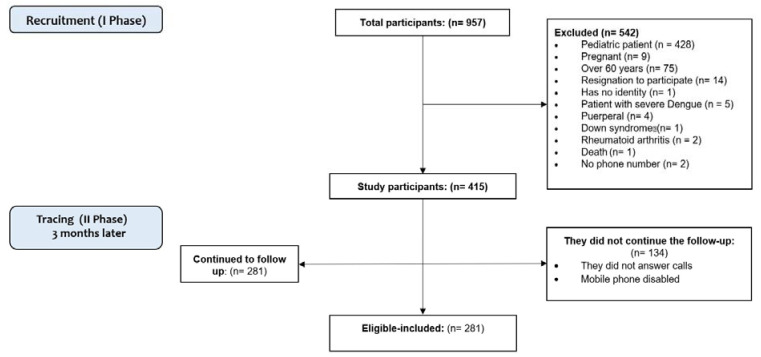
Flow chart of the patients included and excluded.

Participants who had met the dengue case criteria included participants who were between 18 and 60 years of age with a diagnosis of dengue and participants who voluntarily agreed to participate in this study through informed consent (Figure 1). For Honduras, the definition of dengue is a patient with a feverish illness of sudden onset, lasting up to 7 days, with two or more of the following manifestations: headache, myalgia, arthralgias, retro-ocular pain, skin rash, leukopenia, presence or absence of bleeding, and confirmed by a laboratory test, including serology or PCR for DENV.

### 2.2. Exclusion Criteria

Pregnant participants, participants under 18 years of age and over 60 years of age, participants who did not meet the dengue case definition, participants with previous autoimmune or rheumatic diseases, participants without national documentation for identification and without telephone contacts for follow-up, complicated severe dengue cases: severe dengue shock, liver failure, kidney failure, adult respiratory distress syndrome (ARDS), and death (Figure 1), and patients with suspicion of coinfection (e.g., CHIKV) were excluded.

### 2.3. Study Phases

Recruitment Phase (3 months): It was carried out on all participants with a dengue diagnosis treated in the assistance centres or dengue units. In this recruitment phase, there were n = 957 participants, of which n = 542 were excluded by the following criteria: Pediatric patient (n = 428), pregnant (n = 9), over 60 years old (n = 75), resigned to participate (n = 14), no identification (n = 1), patient with severe dengue (n = 5), postpartum (n = 4), participant with down syndrome (n = 1), arthritis rheumatoid (n = 2), death (n = 1), no contact number: (n = 2). Participants who managed to enter this study were n = 415.

Follow-up phase (4 months): All study participants were located by telephone through a private project line available in the informed consent given during enrollment. When we contacted the participant, the WOMAC and DAS-28 questionnaires were applied again through a telephone interview. In this phase, there were participants who continued with the follow-up (n = 281), and there were participants who did not continue (n = 134) because they did not answer the scheduled telephone calls or had their cell phones disabled.

The project included an email in the Gmail account (womac.das28@gmail.com) that allowed answering questions, making comments, or making changes to the telephone number.

### 2.4. Analysis of Data

Obtaining a sample is affected by the statistical application GPower 3.1. We used a multivariate analysis and the T student and Chi-square tests. The instruments were obtained using the Google Forms application. They were entered and subsequently analyzed in the SSPS version 25 program for the MacBook.

### 2.5. Ethical Considerations

This study was conducted under the Declaration of Helsinki. This research’s preparation and execution fully complied with the fundamental ethical principles of autonomy, justice, beneficence, and non-maleficence. Act Number 2019062, approved by the Ethics Committee in Biomedical Research (CEIB) of the National Autonomous University of Honduras (UNAH), meeting on 11 December 2019.

The protocol was approved by institutional authorization in writing from the General Hospital Atlántida, La Ceiba, Atlántida; Manuel de Jesús Subirana Hospital; and Santiago Apóstol Comprehensive Health Center in Yoro. Written informed consent was obtained, and a copy of the informed consent was given to each participant, explaining the objectives of the project and her voluntary participation in the evaluations through questionnaires. The information was obtained from interviews conducted by members of the research team trained in applying rheumatological assessment scales (WOMAC and DAS-28). There were no potential risks to the participant during the process, and they had no direct benefits from the research; however, the information obtained will serve to understand arbovirus diseases and their relationship with chronic degenerative diseases such as osteoarticular involvement in an increasingly exposed and aged population. Upon completion of the follow-up phase, participants who were presented with any health problem associated with rheumatological sequelae will be referred for specialized medical care in the local hospitals.

## 3. Results

The group of participants was made up of 58.8% women and 41.20% men and an average age of 33 years, with 24.59% having completed secondary education; in terms of occupation, housewives are the most affected, with 61.07%, followed by 19.88% farmers and 0.41% transport employees being the least affected by the illness (Table 1).

Based on the results obtained, the inference is made that the functional limitations or symptoms related to daily physical activity post-dengueoccur in a higher proportion in the female sex, as assessed by the WOMAC questionnaire (Table 2). These symptoms or functional limitations when performing daily activities, comparing women vs. men (significantly higher among the firsts, *p* < 0.0001), were limited to pain when: walking (34.81% vs. 5.51%), going up or down stairs (36.46% vs. 8.66%), at night when lying in bed (28.73% vs. 7.08%), when being seated (30.94% vs. 5.51%), and when standing (33.70% vs. 10.23%); Joint stiffness: upon waking (25.41% vs. 10.23%) and during the day (17.67% vs. 0.78%); Difficulty: to go downstairs (28.17% vs. 4.72%), when getting up (56.90% vs. 9.44%), to stand (27.62% vs. 4.72%), to bend down (30.93% vs. 5.51%), to walk (24.86% vs. 3.14%), to get in and out of the car (22.09% vs. 2.36%), to buy (25.30% vs. 4.72%), to get dressed (46.96% vs. 7.08%), to go to bed (22.65% 3.93%), and to do household chores (23.75% vs. 12.59%). Thus, these results showed a higher percentage of post-dengue rheumatic complications or sequelae in females than in males.

Finally, at DAS-28 (Table 3), the total number of patients, in most of whom this questionnaire was assessed (228), showed at least one alteration in 14.91% of the patients, significantly higher among women (22.2%) (*p* = 0.001) (Table 3). Although 9.65% presented between one to ten joints with inflammation or pain, 4.39% presented from 11 to 30, and 0.88% presented more than 30 altered joints, with no significant differences by gender. Assessing each joint specifically for inflammation or pain revealed that the knee was the most affected, with significant differences by gender, especially with pain; from women, 16.67% presented pain at the left knee and 15.87% at the right knee (Table 3). For eight joints, the proportion of those with inflammation was significantly higher among women (*p* < 0.05), whereas for 15 joints, the proportion of those with pain was significantly higher among women (*p* < 0.05) (Table 3).

## 4. Discussion

Arboviral diseases continue to be a significant public health threat in the world, in endemic and non-endemic areas, due to migration and climate change, which have allowed local transmission in non-endemic territories [22,23]. In South-East Asia and Latin America, its burden is significant, with endemic areas presenting periodical epidemics, as is the case of Honduras in Central America [2,24]. The chronic consequences of dengue have been poorly studied, including the rheumatic sequelae and manifestations in different populations [12,13,25,26]. In fact, there are no studies of the follow-up of DENV patients after the acute phase of the disease. In addition, dengue, more than any other arboviral disease, will be the cause of new outbreaks and epidemics in the near future in Honduras and other Latin American countries. During 2019, DENV-1 and DENV-2 were the circulating serotypes (www.paho.org (accessed on 8 November 2022)); nevertheless, it is important to remember that only a small number of confirmed cases of DENV are sequenced, cultured, and serotyped.

The present study suggested that the non-acute DENV affectation, according to the WOMAC questionnaire, was high during the epidemic in Honduras in 2019. Of the 286 participants who continued the follow-up, 63.02% presented with symptoms. Primary joint pain was observed when walking. Comparing arthralgia was more frequently reported by studies in patients with CHIKV, 84.6% [11], as expected as a classical arthritogenic arbovirus, but less assessed and observed with dengue. Unfortunately, until 2022, no studies have used the WOMAC questionnaire to assess patients affected by DENV. However, not in Chikungunya and Zika either. Follow-up of DENV patients is important, based on the current findings.

According to the WOMAC questionnaire, we also refer to the group with the most significant affectation as being female (58.76%) compared with a meta-analysis of the Zika virus, where the cases that reported signs/symptoms of arthritis were primarily women, which represented 66.7% of the participants from 129 patients [27]. In addition, our study estimated an average mean age of 32 years, compared to dengue cases (median age 24.8 years) [19].

Severe joint pain and stiffness are the hallmarks of arthritogenic alphaviruses [9]. Therefore, the results could be obtained that they presented with severe pain when performing the following activities as walking (63.023%), pain when going up or down stairs with (25%), pain at night when in bed (19.80%), pain when sitting (20.45%), and pain when standing (24.02%). Additionally, concerning stiffness, we obtained that joint stiffness upon awakening was 19.15%, together with stiffness throughout the day was 10.71%.

DAS-28 allows us to observe that a significant proportion of women with joint inflammation and pain, which is higher than zero, is, in general terms, unexpected; showing that dengue may also lead to the development of chronic rheumatological findings, maybe in the lower proportion that CHIKV, but still affecting the everyday life of patients and, consequently, their quality of life. This issue should also be assessed in further studies of post-dengue long-term assessment. For example, a study in Mexico in 2017 using the DAS-28 considered the change between the diagnosis and follow-up of patients with CHIKV infection [28]. This study found that DAS-28 was significantly low at follow-up, indicating chronic compromise. Unfortunately, in DENV, such tools as the DAS-28 have not been previously used and published in the literature until 2022.

## 5. Limitations

Due to the COVID-19 pandemic, the delay in data analysis and obtaining results caused offline telephone numbers; we limited the participants’ contact in the second phase of the investigation. Additionally, the drop-out rate of participants is considerably high (134/415), leading to a smaller number of follow-ups study participants, which certainly should be considered in future studies. In addition, baseline (before dengue infection) WOMAC and DAS-28 questionnaire data were not collected, as patients were included after the initial diagnosis of dengue. Although there are many housewives, farmers, and labor population in this study, subgroup analyses are available. Biomarkers would be useful include in the future for such populations, including PCR and certain interleukins, such as IL-6.

## 6. Conclusions

This is probably the first study assessing post-dengue rheumatic manifestations in a follow-up with the WOMAC and DAS-28 questionnaires. These tools show significant compromise after the acute phase of the disease, suggesting the need for follow-up in DENV patients at least during the first four months after acute illness. Further studies on such populations would be interesting, and follow-up assessments in this population are expected after one year and more of DENV.

## Figures and Tables

**Table 1:** Demographic characteristics and time of follow-up in the northern zone of Honduras.

Demographic Characteristics	Sex		Total
	Female	Male	
	n = 244 (58.8%)	n = 171 (41.20%)	n = 415
Age years old (Media ± SD)	33.05 (±11.7)	30.7 (±10.6)	32.10 (±11.35)
Study site			
La Ceiba-Atlántida	166 (68.03%)	110 (64.33%)	276 (66.51%)
Tocoa-Colón	15 (6.15%)	13 (7.60%)	28 (6.75%)
Yoro-Yoro	63 (24.82%)	48 (28.07%)	111 (26.75%)
Schooling			
None	19 (7.79%)	9 (5.26%)	28 (6.75%)
Complete Primary	50 (20.49%)	40 (23.39%)	90 (21.69%)
Incomplete Primary	29 (11.89%)	29 (16.96%)	58 (13.98%)
Completed Secondary	60 (24.59%)	39 (22.81%)	99 (23.86%)
Incomplete Secondary	56 (22.95%)	37 (21.64%)	93 (22.41%)
University	17 (6.97%)	5 (2.92%)	22 (5.30%)
Incompleted University	13 (5.33%)	12 (7.02%)	25 (6.02%)
Occupation			
Administrator and management	0	2 (1.17%)	2 (0.48%)
Farmer	1 (0.41%)	34 (19.88%)	35 (8.43%)
Housewife	149 (61.07%)	0	149 (35.90%)
Trader and seller	15 (6.15%)	17 (9.94%)	32 (7.71%)
Office Worker	8 (3.28%)	1 (0.58%)	9 (2.17%)
Student	21 (8.61%)	19 (11.11%)	40 (9.94%)
Transportation Employee	1 (0.41%)	9 (5.26%)	10 (2.41%)
Production workers	2 (0.82%)	9 (5.26%)	11 (2.65%)
Labourers	0	33 (19.30%)	33 (7.95%)
University and Technical	22 (9.02%)	9 (5.26%)	31 (7.47%)
Customer Service	17 (6.97%)	19 (11.11%)	36 (8.67%)
Others	8 (3.28%)	19 (11.11%)	27 (6.51%)
Time of follow-up Weeks (median)	16	17	16
Minimum time	6	14	6
Maximum time	37	31	37

SD: Standard deviation.

**Table 2:** Symptoms and physical activity participation with the WOMAC questionnaire by sex at the follow-up (four months).

WOMAC Questionnaire	Female n = 165 (58.7%)	Male n = 116 (41.3%)	Total n = 281	*p*-Value ^a^
How much pain is there?				
To go up or down stairs	62 (37.6%)	9 (7.8%)	71 (25.3%)	<0.001
Walk on flat ground	62 (37.6%)	7 (6.0%)	69 (24.6%)	<0.001
To stand	58 (35.2%)	11 (9.5%)	69 (24.6%)	<0.001
To be sitting or lying down	54 (32.7%)	6 (5.2%)	60 (21.4%)	<0.001
At night in bed	50 (30.3%)	7 (6.0%)	57 (20.3%)	<0.001
How much stiffness is there?				
After waking up in the mornings	44 (26.7%)	11 (9.5%)	55 (19.6%)	<0.001
For the rest of the day, after sitting, lying down or resting	31 (18.8%)	4 (3.4%)	35 (12.5%)	<0.001
How hard are you?				
Doing heavy household chores	56 (33.9%)	11 (9.5%)	67 (23.8%)	<0.001
Climbing stairs	51 (30.9%)	6 (5.2%)	57 (20.3%)	<0.001
Crouch down to pick something up	51 (30.9%)	6 (5.2%)	57 (20.3%)	<0.001
Standing	51 (30.9%)	5 (4.3%)	56 (19.9%)	<0.001
Getting up after sitting	47 (28.5%)	7 (6.0%)	54 (19.2%)	<0.001
Downstairs	48 (29.1%)	5 (4.3%)	53 (18.9%)	<0.001
Shopping	45 (27.3%)	5 (4.3%)	50 (17.8%)	<0.001
Getting out of bed	47 (28.5%)	3 (2.6%)	50 (17.8%)	<0.001
Put your socks on	43 (26.1%)	4 (3.4%)	47 (16.7%)	<0.001
Walking on flat ground	42 (25.5%)	3 (2.6%)	45 (16.0%)	<0.001
Sit back and get up from the toilet	42 (25.5%)	3 (2.6%)	45 (16.0%)	<0.001
Take off your stockings	40 (24.2%)	3 (2.6%)	43 (15.3%)	<0.001
Lying in bed	39 (23.6%)	4 (3.4%)	43 (15.3%)	<0.001
Sit	38 (23.0%)	5 (4.3%)	43 (15.3%)	<0.001
Doing light household chores	41 (24.8%)	2 (1.7%)	43 (15.3%)	<0.001
Get in and out of a car	38 (23.0%)	2 (1.7%)	40 (14.2%)	<0.001
In and out of the bathroom	36 (21.8%)	4 (3.4%)	40 (14.2%)	<0.001

^a^ Comparison by χ^2^, female versus male.

**Table 3:** DAS-28 findings. Joints with inflammation and pain at follow-up (four months).

DAS-28 Questionnaire		Female (n = 126) (%)	Male (n = 102) (%)	Total (228) (%)	*p*-Value
Any alteration at DAS-28	Yes	22.22	5.88	14.91	0.001
Number of joints with pain or inflammation	None	77.78	94.12	85.09	0.187
	1 to 10	13.49	4.90	9.65	
	11 to 30	7.14	0.79	4.39	
	31 or more	1.59	0.00	0.88	
Inflammation of joints					
Left	shoulder	0.79	0.98	0.88	0.888
	elbow	0.00	0.00	0.00	-
	wrist	3.17	0.00	1.75	0.069
	MCP-1	2.38	0.98	1.75	0.423
	MCP-2	1.59	0.98	1.32	0.689
	MCP-3	1.59	0.98	1.32	0.689
	MCP-4	1.59	0.98	1.32	0.689
	MCP-5	1.59	0.98	1.32	0.689
	PIP-1	2.38	0.98	1.75	0.423
	PIP-2	2.38	0.98	1.75	0.423
	PIP-3	2.38	0.98	1.75	0.423
	PIP-4	2.38	0.98	1.75	0.423
	PIP-5	2.38	0.98	1.75	0.423
	knee	11.90	1.96	7.46	0.004
	ankle	4.76	0.98	3.07	0.100
Right	shoulder	1.59	1.96	1.75	0.831
	elbow	2.38	0.00	1.32	0.117
	wrist	3.17	0.00	1.75	0.069
	MCP-1	3.97	0.00	2.19	0.042
	MCP-2	3.17	0.00	1.75	0.069
	MCP-3	3.17	0.00	1.75	0.069
	MCP-4	3.17	0.00	1.75	0.069
	MCP-5	3.17	0.00	1.75	0.069
	PIP-1	3.97	0.00	2.19	0.042
	PIP-2	3.97	0.00	2.19	0.042
	PIP-3	3.97	0.00	2.19	0.042
	PIP-4	3.97	0.00	2.19	0.042
	PIP-5	3.97	0.00	2.19	0.042
	knee	9.52	1.96	6.14	0.018
	ankle	4.76	0.98	3.07	0.100
Pain of joints					
Left	shoulder	3.97	0.98	2.63	0.161
	elbow	4.76	0.00	2.63	0.026
	wrist	7.94	0.98	4.82	0.015
	MCP-1	4.76	0.98	3.07	0.100
	MCP-2	4.76	0.98	3.07	0.100
	MCP-3	4.76	0.98	3.07	0.100
	MCP-4	4.76	0.98	3.07	0.100
	MCP-5	4.76	0.98	3.07	0.100
	PIP-1	5.56	0.98	3.51	0.062
	PIP-2	5.56	0.98	3.51	0.062
	PIP-3	5.56	0.98	3.51	0.062
	PIP-4	5.56	0.98	3.51	0.062
	PIP-5	5.56	0.98	3.51	0.062
	knee	16.67	2.94	10.53	0.001
	ankle	0.00	0.00	0.00	-
Right	shoulder	4.76	1.96	3.51	0.253
	elbow	5.56	0.00	3.07	0.016
	wrist	8.73	0.00	4.82	0.002
	MCP-1	6.35	0.00	3.51	0.01
	MCP-2	6.35	0.00	3.51	0.01
	MCP-3	6.35	0.00	3.51	0.01
	MCP-4	6.35	0.00	3.51	0.01
	MCP-5	6.35	0.00	3.51	0.01
	PIP-1	6.35	0.00	3.51	0.01
	PIP-2	6.35	0.00	3.51	0.01
	PIP-3	6.35	0.00	3.51	0.01
	PIP-4	5.56	0.00	3.07	0.016
	PIP-5	6.35	0.00	3.51	0.01
	knee	15.87	2.94	10.09	0.001
	ankle	0.00	0.00	0.00	-

MCP: metacarpophalangeal joint; PIP: proximal interphalangeal. Bold, significant values.

## Data Availability

The data presented in this study are available upon request from the corresponding author.
